# Leadership Competence and Psychosocial Safety Climate Implementation in an Evolving School Work Environment

**DOI:** 10.3390/ijerph23050573

**Published:** 2026-04-29

**Authors:** Stefano Cataloni, Darryl Forsyth, David Brougham, Kaye Thorn

**Affiliations:** School of Management and Marketing, Massey University, Auckland 0632, New Zealand

**Keywords:** leader competence, school leadership, psychosocial safety climate

## Abstract

**Highlights:**

**Public health relevance—How does this work relate to a public health issue?**

- Teachers’ psychological health is a critical occupational health concern, with school leadership shaping psychosocial safety climate (PSC) and psychosocial risk exposure.
- Leadership competence (encompassing leaders’ skills, knowledge and behaviours) supports the implementation of positive PSC and protects psychological health and safety.

**Public health significance—Why is this work of significance to public health?**

- Leadership competence offers a novel and complementary theoretical lens for understanding PSC implementation within workplaces and its implications for working conditions.
- Leader competence influences perceptions of PSC and contributes to teachers’ psychosocial work environment.

**Public health implications—What are the key implications or messages for practitioners, policy makers and/or researchers in public health?**

- This study highlights the importance of developing actionable leadership competencies to improve teachers’ psychological health and safety.
- The Leadership Competence–PSC Framework offers a practical tool for building leader capability across specific competencies and PSC domains.

**Abstract:**

The purpose of this study is to examine how the leadership competence of school leaders supports the implementation of psychosocial safety climate (PSC) within an educational workplace. While recent studies have considered how various leadership styles influence PSC, the processes through which school leaders at different levels enact and develop PSC in practice continue to receive limited attention. This study addresses this gap through a qualitative case study at a school in Aotearoa, New Zealand, which employed a sequential data collection process comprising 26 interviews and three focus groups. This investigation found that exemplary leadership, overcoming complexity, and multiskilled leadership are pivotal competencies that enable PSC implementation within a school setting. More broadly, we discuss how these key leadership competencies facilitate the development of policies, practices, and procedures that promote teachers’ psychological health and the four domains of the PSC framework. Finally, we propose a Leader Competence–PSC Framework as a practical tool for investigating and evaluating school leader competence across specific PSC domains.

## 1. Introduction

This study explores how leadership competence enables school leaders to implement a positive psychosocial safety climate (PSC) in an Aotearoa New Zealand school setting. Dollard and Bakker [1] (p. 580) defined PSC as the “policies, practices, and procedures for the protection of worker psychological health and safety (H&S),” involving a climate free from psychological harm. An organisation’s PSC reflects the working conditions that affect employees’ psychological health and engagement [2] and largely depends on the policies, practices and procedures that management designs and implements [3]. PSC is strengthened when organisations and leadership create a psychologically safe environment and positive work culture [1].

In relation to leadership, the literature generally focuses on how a positive PSC setting enables effective leadership and management practices, rather than examining how leadership impacts PSC [4]. In general, research suggests that leadership quality can be determined by an organisation’s PSC [5] and that it positively impacts leadership behaviours [6,7]. However, more recently, research has begun to investigate the reverse relationship—the impact of leadership on PSC, through Laloo et al.’s [4] recent call for such research. These calls for greater attention to the impact of leadership on PSC have prompted several quantitative studies to examine how different leadership styles influence PSC [8,9,10,11], collectively demonstrating that PSC is shaped not only by management policies, practices and procedures but also by different leadership styles.

However, while existing PSC literature in this area sheds light on how various leadership styles influence PSC [8,9,10,11], it has not explored the relationship between school leadership competence and PSC. Examining this relationship may offer a complementary and additive perspective of how leaders’ specific skills, knowledge and behaviours can support PSC implementation within school settings. As Wong et al. [12] posited in relation to leadership more generally, focusing exclusively on leadership styles can obscure leader behaviours that are critical for drawing richer, more contextual conclusions. Despite leadership being a conceptually broad and complex construct, the narrow, typically quantitative focus on leadership styles in the PSC literature may constrain deeper, more nuanced understandings of the relationship between leadership and PSC. This view is echoed in a recent review of leadership literature by Liden et al. [13], who conclude that future scholarship should seek to identify profiles of leader behaviours that vary with situational factors and follower needs—a process best achieved through a qualitative, inductive lens.

Hence, our study seeks to provide rich, contextually connected insights into how and why specific leadership competencies enable the implementation of PSC within an educational setting. Exploring leadership competence in relation to PSC shifts the focus from categorising leaders through leadership-style frameworks to examining competencies that recognise the complex, contemporary challenges prevalent in schools. This approach aligns with Liden et al.’s [13] position that leadership is better understood by investigating profiles of leader behaviours and skills that vary with situational factors and follower needs, rather than relying on and being constrained by predefined leadership styles. Our study conceptualises leader competence as “the knowledge and skills necessary for effective leadership… whether due to natural talent or developed skill (or more often both)” [14] (p. 350), and that competence reflects what a leader can do, often through the manifestation of behaviours [15]. In response to the gaps in the literature identified above, this study investigates the following research question:

How does leadership competence enable school leaders to implement PSC?

## 2. Literature Review

### 2.1. School Leader Competence

School leaders play a critical role in schools [16] and are required to deliver on an extensive range of tasks and responsibilities [17]. While there is no single agreed-upon list of competencies that leaders require to perform their roles effectively, job descriptions suggest they need a wide range of skills and qualities [18]. This breadth of responsibility is also evident in Robinson et al.’s [19] meta-analysis, which identified building relational trust, integrating educational knowledge, and solving complex problems as necessary and interrelated leadership capabilities. Furthermore, school leaders must be capable of leading teams in volatile, uncertain, complex, and ambiguous settings, amid increased compliance and workload demands [20].

Multiple studies have reinforced that strong interpersonal skills are essential in school leadership roles. For instance, good awareness [21], empathy [22], strong relationships [23], and communication [24] positively impact working conditions. Similarly, Brady and Wilson [25] highlighted that leaders must understand the needs and wants of staff, while Acton and Glasgow [26] asserted that teachers should feel heard, valued, and supported—both socially and professionally by leaders. Conversely, teachers can experience a loss of trust and excessive stress when leaders demonstrate poor communication and interpersonal skills and fail to follow through on decisions [27,28].

School leader support is well-documented in the literature as a key leadership competence that positively impacts teacher wellbeing. For instance, it is positively associated with teacher agency, resilience, and commitment, and may also affect teacher quality [27,29]. Leader support enables teachers to perform their roles confidently as they confront and overcome the various challenges of their job. This support also allows leaders to monitor teacher workloads and foster positive work environments, enabling harmonious relationships between leaders and staff and encouraging teachers to learn and improve their practice [27]. Hence, school leaders play an integral role in supporting and strengthening teachers’ capabilities, which, in turn, can lead to higher teaching and learning outcomes [30].

Teachers and leaders also view leader support as a pivotal part of a leader’s role, though their perspectives differ on what constitutes it [31,32]. For example, some school leaders report feeling responsible for supporting the wellbeing of their staff [33]. Another survey of leaders found that coaching and guiding people are core to their job, while teachers viewed leadership support as facilitation and as recognising staff’s achievements and contributions [31]. These different perspectives may have implications for which leadership competencies school leaders practise and which ones actually positively impact teachers’ perceptions of PSC.

### 2.2. Psychosocial Safety Climate (PSC)

Psychosocial safety climate (PSC) is an organisational resource specifically concerned with employees’ psychological health [34]. It consists of four domains: (1) management commitment and support, (2) management priority, (3) participation in health and safety (H&S) activities and policy, and (4) organisational communication [35]. PSC can be strengthened across these four domains by identifying potential risks and implementing policies, practices and procedures that improve employees’ psychological health [36]. Furthermore, these preventive and supportive measures increase employee participation and awareness of staff psychological H&S issues [6].

The management commitment and support domain focuses on how quickly and decisively management takes action to rectify issues relating to employee psychological H&S [37] and includes the degree to which management prevents or mitigates psychosocial risk factors [2]. The management priority domain focuses on the extent to which management prioritises employees’ psychological health relative to the organisation’s production or productivity imperatives related to growth [38]. The organisational participation domain concerns the involvement of members at different levels of the organisation and across different interest groups (e.g., management, employees, unions, and H&S representatives) in ensuring that employee psychological H&S is protected [39]. Finally, the organisational communication domain concerns the extent to which organisations and leaders communicate psychological H&S matters to employees [23] and includes how well employee concerns are heard and acted upon [37].

Biron et al. [40] described a positive PSC setting as reflecting management priorities for stress prevention and a participative culture, in which various stakeholders are involved in psychological H&S decision-making. Leaders are instrumental in shaping organisational culture and have the authority to develop and implement policies, practices and procedures related to employees’ psychological health [3]. Therefore, leaders who proactively identify workplace psychosocial risks, mitigate excessive work demands and ensure that work is appropriately challenging are more likely to develop a strong PSC [41]. Conversely, within a school context, a lack of coordination and resourcing initiated at higher levels in schools was found to undermine employees’ self-regulation by interfering with task performance, personal development, job stability, and work–life balance [42]. Similarly, Yulita et al. [34] reported that principals who enacted managerial support (doing) rather than only espousing PSC (saying) made teachers feel safer taking appropriate measures to protect themselves from psychological injury. Moreover, the Malaysian study noted that principals can strengthen PSC by valuing psychological health and by transforming espoused PSC into enacted policies, practices, and procedures.

As mentioned in the introduction, recent research has examined the impact that PSC has on leadership. For example, organisational PSC is positively related to constructive leadership but negatively related to laissez-faire leadership as constructive leadership appears to mitigate workplace bullying and promote higher PSC, whereas laissez-faire leadership is more likely to enable bullying [6]. Similarly, research on Swedish engineers has shown that positive PSC interventions lead to improvements in leadership behaviours [7], reinforcing PSC’s implications for leadership.

While the above studies have shown that strong PSC is associated with quality leadership and specific leadership styles, there is a paucity of literature investigating the opposite relationship—leadership’s potential impact on PSC [39]. As discussed earlier, most research to date investigating this direction of impact has focused on leadership styles. Previous studies include Loh et al. [9], who demonstrated that PSC leadership, but not transformational leadership, is strongly associated with positive PSC. A Greek study also reported that servant leadership could be an effective leadership style for achieving stronger PSC [43], while Dollard and Jain [44] claimed that ethical leadership might mitigate corrupt workplace practices and lead to a stronger PSC. However, not all studies investigating the impact of leadership styles on PSC report a positive relationship. While Laloo et al. [8] claimed that authentic leadership appears to positively impact PSC, ethical, transformational, and transactional leadership styles were found to have no impact on PSC. The present research provides a complementary and additive perspective on the potential impact of leadership on PSC by investigating how specific leadership competencies facilitate PSC implementation in a school setting.

## 3. Context

The participating secondary school, with approximately 70 staff and 1000 students, has four teams, each comprising two line managers and 13 staff members (including one team assistant). Despite being line managers, staff refer to them as mentors and senior leaders, as they are pivotal members of the school’s senior leadership team. Line managers have curriculum oversight for their teams and are responsible for integrating the school’s teaching and pastoral objectives into their teams’ settings. Collectively, this group of staff and line managers constitutes a professional learning community (PLC) that works collaboratively on a daily basis, and meets formally weekly to facilitate staff interaction, share accomplishments, reflect on practice and student outcomes, and develop strategies to address issues across different subject areas [45].

## 4. Materials and Methods

This research presents an explanatory case study, which is ideal for explaining ‘how’ and ‘why’ interactions and events between phenomena occur [46]. Data were gathered sequentially through semi-structured interviews and focus groups over five months, creating conditions for robust qualitative research [47] and case study research [48]. The content of the focus groups was meaningfully informed by the semi-structured interviews, thereby identifying and filling knowledge gaps and facilitating data triangulation [49]. An interview pilot study was conducted with five participants (mostly teachers) to refine the questions and ensure coverage of the topic [50] before field data were collected. The study was reviewed and approved by the University’s Human Ethics Committee. Before any interviews or focus groups were conducted, written consent was obtained from participants. All interviews were transcribed and anonymised by the researchers.

The sample frame comprised teachers, school leaders, and support staff (e.g., school counsellors) who could speak extensively about school leadership and PSC. Since the study focused on school staff, no data on students were collected, as this was outside the study’s scope. For adequate representation, we captured a balanced cross-section of leaders and staff from across the school’s four teams, comprising two leaders, 12 teachers and one team assistant. Staff such as receptionists, IT technicians, the school’s executive administrator, and finance personnel were excluded because they do not work as closely with teachers and are less knowledgeable about PSC in relation to daily teaching and leadership practices.

Participants’ demographic data encompassing gender, age, ethnicity, team membership, length of tenure at the school and in the profession overall, employment status (comprising full-time and part-time), and weekly hours spent completing work were collected, as depicted in Table 1. Importantly, participants were broadly representative of the school’s general staff. For example, 23% of participants were school leaders, which is similar to the 20% of the school’s total staff who are leaders. Likewise, the participant gender distribution was 65% female and 35% male, closely matching the school’s 67% female and 33% male split.

Purposive sampling is effective at capturing varied viewpoints, particularly when involving different groups [51], which was deemed the most appropriate for the present study, given the relatively semi-autonomous functioning and leadership of each team. The interview sample size was guided by the principle of theoretical saturation. This involved increasing the sample size until no new data emerged [52]. Although data saturation appeared to have been reached by the 21st participant, data collection continued for 5 additional interviews to ensure saturation and to facilitate representativeness across teams. A total of 26 semi-structured interviews were conducted, comprising 19 teachers, 5 senior leaders, and 2 support staff. Participants were first recruited through an invitation email sent on our behalf to all staff, ensuring that every leader and staff member across the school’s four teams received the opportunity to participate and enabling proportional representation. The email outlined the research objectives and detailed participants’ potential involvement in both the interview and focus groups. Before commencing the data collection, the first author was introduced to the entire staff at an all-staff meeting by the school’s principal, during which the research objectives and participants’ involvement in the investigation were discussed. This was soon followed by an additional all-staff follow-up email, which prompted further interest from a wider range of participants (roles, seniority, and team affiliation).

The purpose of the semi-structured interviews was to develop an in-depth understanding of the key phenomena and the potential links between leadership competence and PSC. First, the interviews sought perceptions on leadership competence. For instance, participants were asked, “Why do you think it is important for teachers to have competent leaders?” Second, questions focused on the school’s PSC. For example, “Do you think leaders prioritise and support the psychological health of staff?” Third, questions prompted discussion of the potential link between leader competence and PSC. For example, “Do you have views on how leader competence may contribute to making your school a psychologically healthy and safe workplace?”

Stage two comprised three focus groups (two with teachers and one with school leaders). The objective was to develop a firsthand understanding of the school’s leadership functions related to PSC, particularly at the line-management level. Participants who had taken part in the interviews were invited to join the 55-min focus groups. Including the same participants in the focus groups enabled them to reflect on the earlier interview and on their recent experiences related to leadership and PSC, and to speak within a group setting. The two data types facilitated triangulation while also reducing respondent [47] and researcher bias [53] by comparing specific findings from one data source with those from another. The focus groups also served as a form of member-checking, in which data analysed from the interviews could be checked and affirmed by participants in subsequent focus groups. A predetermined sample size was not set for the number of focus group sessions, as the central purpose of focus groups was not to develop new themes or achieve saturation but to deepen understanding of the themes identified through interview data.

The authors were not associated with the participating school prior to the investigation. The first author is a former school teacher and brought extensive contextual knowledge of the Aotearoa New Zealand schooling system, along with prior assumptions that were identified and considered throughout this reflexive research process. Several measures were adopted to facilitate researcher reflexivity, which, as Morse [54] claims, can mitigate researcher bias and enhance a study’s credibility. While bias cannot be completely eliminated in qualitative research, important safeguards that enable research reflexivity can reduce the misinterpretation of data [55]. For instance, during interviews Merriam and Tisdell’s [56] advice was followed to maintain a neutral stance and avoid leading questions that might reflect the researchers’ assumptions and influence participants’ responses. Additionally, the three-month time lapse between analysing the interview data and conducting the focus groups enabled reflexive practice, as we could identify and critique key interview findings and compare them with those from the focus groups.

Data analysis followed Braun and Clarke’s edict [57]: (a) familiarisation with the data; (b) generation of codes; (c) combining codes into themes; (d) reviewing themes; and (e) determining the significance of the resulting themes. As data were collected, separate coding systems were developed for each data collection stage, followed by multiple revisions, to ensure that the codes were a credible interpretation of the data [51]. Initial coding involved labelling data using distinct and defining features, such as keywords and participants’ phrases, to identify overarching patterns [58]. An iterative approach of renaming and documenting code tables ensured that code labels accurately reflected the data they represented.

NVivo 15 software was used to manage data and develop codes across the datasets. Coding tasks, such as identifying frequencies and applying a single code across similar pieces of text and data, are more manageable using computer-assisted software such as NVivo [59]. NVivo was particularly useful for identifying narrow-scope codes that required merging and for highlighting overlooked codes elevated in the coding hierarchy. Commonalities across codes from the different data were identified and used to develop themes. For example, separate codes related to ‘exemplary leadership’ were derived deductively from data collected through interviews and focus groups.

The resulting themes and subthemes were reviewed to check that they were a credible representation of the data. The identification and explanation of patterns and interconnections [60] highlighted the relative importance of the various themes and subthemes and revealed their links. Overall, the two data sources facilitated triangulation, further substantiating the themes [48], while the analysis enabled critical reflection and deep engagement with the data [57]. In addition, the authors engaged in regular peer review throughout the data analysis to ensure a credible and trustworthy interpretation of the data, particularly during theme development.

## 5. Results

Various acronyms are used to denote the different types of data used in this study: P refers to teaching and support staff Participants and their assigned numbers, while SL relates to School Leaders and their assigned numbers. TFGP relates to Teacher Focus Group Participant and their assigned numbers, while SLFG refers to Senior Leader Focus Group and their assigned number.

### 5.1. Exercising Leadership Competence Within a School Setting

An inductive analysis of the data revealed three primary themes: *exemplary leadership*, *navigating complexity* and *the multiskilled leader*. These primary themes and their respective subthemes represent the competencies identified as having the greatest impact on enabling PSC implementation.

#### 5.1.1. Exercising Exemplary Leadership Within a School Setting

Findings indicated that leaders who practise exemplary leadership were more likely to be perceived by participants as possessing competencies that have positive outcomes for PSC implementation. The exemplary leadership primary theme consists of three subthemes: trust, ‘walking the talk’, and leader accessibility. Participants considered these three competencies to be essential for demonstrating exemplary leadership and, in turn, enabling PSC implementation.

**Trust Subtheme.** An essential leadership competency that participants associated with exemplary leadership is trust. Participants emphasised its importance because of how it makes teachers feel supported in their roles, especially when facing challenging situations or needing leadership assistance. However, teachers stressed that they would seek support or voice their concerns to a leader only if their trust in that leader was strong. TFGP5 noted:

Whether it’s to do with the kids, organising trips or anything such as workload, but also personal struggles, such as mental health or something else… hopefully the relationship that you have with your leaders is one of trust—that you know you can go to them and you know you’re going to feel supported.

Line managers also emphasised the importance of establishing trust with their direct reports. As the following quote emphasises, trust may also determine the degree to which a teacher feels secure enough to confide in a leader.

The first thing that you need to establish is that you are trustworthy and that you have empathy and understanding for where they [teachers] come from and what they struggle with. If you don’t display those things, they’ll never become comfortable with you (SLFGP6).

As such, teachers’ perceptions of team and individual-level PSC may have depended on their trust in their line managers. This indicates that, even with positive perceptions of school-level PSC, team and individual-level PSC may be lower when trust between teachers and line managers is low. Since teachers spend extended periods with their assigned teams, perceptions of team PSC are important, as these perceptions may impact their day-to-day working conditions.

**Walking the Talk (Leader Credibility) Subtheme.** Data indicated that leader credibility can also shape perceptions of exemplary leadership. This usually involved leaders addressing some of the challenges teachers face. These experiences may not only have helped leaders understand some of the challenges teachers encounter in their roles, but also encouraged teachers to approach them when facing similar issues, as they were more confident that their leaders were ‘in touch’ with common classroom challenges.

Conversely, when teachers’ perceptions of leader credibility were poor, they were often viewed as less competent. For instance, when leaders were unable to demonstrate competence in a specific area of their role, perceptions of their leaders’ credibility decreased. P5 gave this example:

I had these kids that were absolutely horrendous… There wasn’t a proper intervention on the leaders’ part, which made me question whether they were competent in this area. It told me that in the future, if anything is happening, you aren’t the person I want to go back to. I need to find somebody else because I wasn’t happy with the way they dealt with it.

Therefore, perceptions of credibility may have influenced whether a teacher sought support in a similar challenging situation in the future. However, not seeking the leader’s support could have implications for the teacher’s psychological H&S, as it may lead the teacher to absorb all the pressure in addressing the challenge alone.

**Leader Accessibility Subtheme.** This subtheme also reflects exemplary leadership, as most participants valued leader accessibility. Participants appeared to appreciate leaders who were accessible, particularly when urgent assistance was needed. A teacher explained that leaders who are accessible are also usually going to be more approachable and ready to support staff: *“They’re* [leaders] *really approachable, the door’s always open, and you can talk to them. They’ve got time to address any issues and are really good at working through problem-solving* (P14).*”*

Leaders also acknowledged the importance of being accessible, so that teachers typically felt well supported and cared for. As the following quote suggests, having accessible leaders is likely to positively impact teachers’ psychological H&S: *“If you’ve got a leadership team who are accessible, seem to care and know their staff—I see that as the foundation for an emotionally and physically safe worksite”* (SL5).

#### 5.1.2. Navigating Complexity

This primary theme concerns leaders’ needing to understand and address complex issues prevalent in schools. It comprises three subthemes: handling of pastoral issues, addressing misbehaviour, and managing pedagogical pressures. Given the nature of these issues, participants considered them essential competencies in a constantly demanding and continuously evolving school environment.

**Handling of Pastoral Issues Subtheme.** This subtheme underscores the importance of school leaders being aware of the daily pastoral challenges students face at home and at school. Most of these challenges appeared to stem from broader societal issues that are increasingly prevalent in educational settings. Some of the pastoral challenges that were impacting schools are outlined by SL3:

I think our classrooms mirror society… with lockdowns and financial crises, I think socialisation has taken a little bit of a whack… I’ve noticed that more parents are struggling at the moment, and perhaps for some of our students, school is the most stable part of their life—an opportunity to connect with peers.

However, the data also suggested that school leaders may be able to mitigate the impact of some of these pastoral challenges on student learning and, consequently, on teachers’ psychological H&S.

We’re in the middle of a mental health crisis, right? … There are tons of kids out there who are going through this… They [line managers] have a really tough job. Well, especially if you’ve got difficult kids, you have team leadership, and then they take care of it or attempt to. But sometimes they get some really impossible kids or really impossible situations to deal with, so I commend them for everything they have to do (P4).

Follow-up question: “*And do you feel as though you’re always supported by them?*”

Participant 4: “*Yes, great in all teams I’ve been in. Particularly, my leader now does so much of that behind-the-scenes work.*”

Teachers explained that leaders regularly supported students with pastoral issues, spoke to parents by phone, or assisted teachers with pastoral challenges. Competence in pastoral care appeared to partly relieve classroom teachers of this pressure, allowing them to focus on classroom teaching. Consequently, data indicated that leaders needed skills that minimise the negative impacts of these pastoral challenges on student learning and the potential flow-on effects on teachers’ psychological H&S.

**Addressing Misbehaviour Subtheme.** Ongoing behavioural issues can impact teachers’ psychological H&S, often requiring prompt support from school leaders. Teachers recognised the impact that student misbehaviour had on student learning and their capacity to engage in effective teaching, which may also impact their psychological H&S. These perceptions are supported by a comment made by an experienced line manager: *“I think definitely behaviour within the classroom is something that perhaps has become more challenging over time”* (SL5).

Teachers highlighted that they rely on leaders’ support when behavioural issues arise. For example, P9 said, *“You might have a student behaviour issue, and sometimes there’s a problem. When you don’t have the support in the classroom, then we look for our leaders to come and help out.”* Leaders who effectively addressed student behavioural issues were not only valued by teachers but were also perceived as possessing a specific competency to address problems that teachers believed were growing and impacting teaching and learning. Competence in addressing misbehaviour appeared to enable leaders to provide timely and effective interventions, which positively impacted perceptions of management commitment and support for teacher psychological H&S.

**Managing Pedagogical Pressures.** Participants cited large class sizes and a growing proportion of students requiring learning support, therefore heightening the need for leaders to be competent in supporting teachers in addressing these challenges. Evidence indicated that pedagogical challenges were impacting teaching at the school. SLFGP2 spoke about the increased learning needs of students:

Now, at least 10% of students either have severe needs or learning needs such as gaps in learning or neurodiversity. So, as classroom teachers—especially as a beginning teacher, how do you deal with that, and with 34 kids in your class?

The quote above suggests that pedagogical pressures are increasing and that teachers require more support. Because schools are under-resourced, this type of support is often provided by school leaders. A teacher shared the following perspective on the pedagogical pressures they regularly faced in the classroom:

In many classes, there are over 30 kids, where you’ll have kids who may have a learning challenge of some kind, such as a processing challenge, where they’re a lot lower than where they’re supposed to be… Then there’s the kid who can’t speak English. Did I say a word to them? No, because I was too busy dealing with the other kid… The kids are bringing something new to you every day…Things pop up, so I’m always like, “OK, where’s the leader? What do I do with this?” …You really do have to have that higher-up person to go to (P17).

These findings indicated that teachers need school leaders who are not only aware of the pedagogical pressures they face but can also promptly intervene and provide appropriate support. Such skills and knowledge may also help ensure that current and future policies and procedures take into account pedagogical pressures while also facilitating management commitment and support.

#### 5.1.3. The Multiskilled Leader

This primary theme reflected participants’ perspectives that school leaders needed to be multiskilled across a variety of skills and behaviours to exercise leadership competence. While participants did not provide a set of competencies that defined a competent leader, participants believed that individual school leaders require a range of leadership skills to effectively implement PSC in school settings. The multiskilled leader primary theme comprises interpersonal skills, proactive problem-solvers, and ‘the experts’ subthemes.

**Interpersonal Skills Subtheme.** Participants agreed that leaders require interpersonal skills to effectively support teachers and address the numerous challenges prevalent in schools. The degree to which participants value interpersonal skills may also have implications for how school leader competence impacts perceptions of PSC. As SLFGP6 noted:

I think in the past, you could have gotten away with a leader who was quite confident in subject knowledge. Now you need a competent leader in soft skills… You need to be able to deal with different staff in different ways… What works for one doesn’t work for the other.

The leader’s quote also recognised that interpersonal skills enable leaders to respond to current school challenges, necessitating a greater focus on developing leaders’ interpersonal skills to support teachers and potentially mitigate threats to teachers’ psychological H&S. As such, strong interpersonal skills may provide teachers with various forms of care, which may encourage teachers to seek support from their leaders.

Communication skills and empathy were also commonly regarded as interpersonal skills that enable the implementation of PSC. Participants often viewed these attributes as prerequisites for building trust and fostering organisational communication with leaders:

I put communication skills and empathy over and above just about everything, I think—because the rest follows. So if you can connect… that communication aspect, if you feel that you can go and chat to a senior leader or you can go and have a transparent conversation with them and they don’t close the door behind them or can make you feel at ease, to me that’s a really good leader (P20).

The perspective above highlights the relational nature of leadership and its impact on teachers’ perceptions of PSC. When leaders exercise effective communication and empathy, they create conditions and opportunities that are conducive to dialogue that can mitigate psychosocial hazards.

**Proactive Problem-Solvers Subtheme.** Teachers indicated that competent leaders can address issues proactively and decisively. This subtheme presents a clear link with the addressing misbehaviour and the dealing with pedagogical pressures subthemes. However, addressing misbehaviour and pedagogical pressures requires leaders to possess context-specific knowledge and expertise. Conversely, the proactive problem-solvers subtheme focuses on leaders possessing skills that prevent issues from arising or to address them immediately when they occur. For instance, P7 gave this perspective:

Competent leadership means that they’re [leaders] not only able to resolve problems but also have things in place that stop these problems from happening in the first place. Being able to handle things and being proactive—taking the necessary steps to do whatever it means to be done.

Teachers appreciated leaders who not only listened to their concerns but also responded promptly with appropriate measures to minimise the potential impact on those involved. Conversely, failing to provide timely commitment and support through proactive action may exacerbate the impact the issue has on teachers’ psychological H&S.

**‘The Experts’ Subtheme.** Participants perceived competent leaders as teaching and learning experts. This expertise may have helped address difficulties a teacher is experiencing that, if left unaddressed, could lead to adverse health outcomes. The ‘experts’ subtheme has clear links with the pedagogical pressures subtheme. However, while the pedagogical pressure subtheme relates to leaders’ knowledge of classroom challenges arising from students’ diverse learning needs and large class sizes, the experts subtheme focuses on leaders’ teaching and learning expertise that they could use to mentor or coach other staff. As P3 mentioned:

They keep up to date with all the research, and so they’re really knowledgeable, and they’re really excited and dedicated. I do think that does feed through because you’re more likely to be like—“I’m excited too…” And so, I just think it’s that natural knock-on effect potentially.

Pedagogical expertise was identified as pivotal for supporting beginning teachers or those teaching a new subject. As P5 stated, *“Some leaders are really good at helping, if you’re struggling with what you want to teach.”* Therefore, this subtheme exemplifies the provision of teaching and learning expertise, especially for teachers who require additional support, which may mitigate some of the pressure they experience when teaching a new subject.

## 6. Discussion

This research found that teachers only sought leader support when trust was present in the relationship. The literature has also emphasised that trust is vital for teacher–leader relationships [33] and improving PSC [3] since the management commitment and support domain reflects perceptions of leaders’ reliability and trustworthiness [6]. When employees receive a signal from their leaders that they can be trusted and that it is safe to practise safety behaviours [12], teachers feel safe seeking their leaders’ support even in challenging situations. This is because, when leadership takes employee concerns seriously, employees are more likely to develop greater trust in their leaders [61]. Therefore, leaders should focus on developing trusting environments [62] so that teachers feel safe suggesting ideas and raising issues, including those related to psychological H&S, which in turn, may encourage organisational communication and participation.

Conversely, when trust is perceived as weaker within team settings, it not only shapes perceptions of leaders’ competence but also team and individual-level PSC. Given that teachers spend most of their time in their team settings (e.g., assigned department, house, and/or professional learning community), experiencing distrust in line management may impact teachers’ day-to-day working conditions. This is because trust functions as a social resource essential to the successful implementation of policies, practices, and procedures [3], where leaders signal safety and teachers feel safe to engage with them in matters relating to psychological H&S. In less-trusting environments, employees are discouraged from seeking support because they perceive their psychological H&S as not valued by their leaders [63]. As such, weaker perceptions of PSC may have negative implications for teachers’ psychological H&S, as they are more likely to rely on maladaptive coping strategies when dealing with issues alone. This insight demonstrates how diminished trust in line managers can create barriers to teachers’ access to valuable job resources (e.g., line management, collegial support, and organisational communication), therefore hindering the implementation of PSC at the team and individual levels. Hence, greater emphasis should be placed on leaders’ trustworthiness as a foundational competency that underpins credibility, strengthens relationships, and enables open communication, which is critical for a positive PSC environment.

Perceptions of leader credibility can depend on employees’ having observed their leaders’ competence in the relevant area [64]. Relatedly, teachers in the present research reported a greater willingness to seek assistance from line managers who had demonstrated the ability to address specific issues, as this often led to more effective outcomes. This finding aligns with Mellor and Webster’s [65] claim that an intervention’s success often rests on the leader’s credibility. Thus, teachers’ perceptions of individual and team-level PSC can be influenced by evaluating their leaders’ credibility based on the success of prior interactions and decision-making. Furthermore, leaders who regularly experience the realities of classroom teaching are more likely to remain attuned to the demands of the teaching role [66], enabling them to recognise teachers’ concerns and implement effective interventions that mitigate psychosocial hazards. Accordingly, this research shows that leader credibility influences teachers’ willingness to engage in organisational communication and the likelihood that the interventions leaders implement will successfully mitigate psychosocial hazards.

Findings on leader accessibility align with Potter et al. [67], who reported that ‘open-door’ policies enable employees to approach line managers with concerns, potentially facilitating organisational communication and participation. Similarly, the present research found that most leaders promote leader accessibility through an ‘open-door’ approach, which may increase upward communication. Accessible leaders foster a climate of safety for teachers [68], enabling staff to report threats before they escalate further [69]. Leader accessibility also made leaders more responsive to teachers’ concerns, which they perceived as prioritising psychological H&S. These behaviours were most evident in team settings, where teachers often worked alongside their line managers, enabling close and meaningful interactions. These findings may support those of Laloo et al. [8], who found that passive-avoidant leadership, characterised by unresponsive and inaccessible behaviours, is negatively associated with PSC, whereas PSC-oriented leaders enact positive practices [9], such as being accessible and promptly addressing teachers’ psychological H&S concerns.

The increasing social and economic challenges that schools face and their impact on learning and teacher practice [25] were also highlighted by participants, who emphasised that leaders must be knowledgeable and skilled at navigating complex issues. External factors create additional organisational demands that can adversely affect working conditions, health, and PSC [70]. While teachers are usually the first responders to most pastoral and behavioural matters, they rely on their leaders’ competence when more complex issues arise. However, increased pastoral care responsibilities and growing student misbehaviour can increase workloads and role ambiguity, both of which are potential psychosocial hazards [71]. Since PSC buffers the impact of excessive job demands [68], this research demonstrates that leaders who mitigate pastoral and behavioural issues can relieve or prevent pressures that teachers face in the classroom. Therefore, the extent to which a complex pastoral or behavioural issue affects teachers’ psychological H&S may depend in part on the leader’s ability to address it before it escalates. Conversely, when such issues are not adequately addressed, this burden may fall entirely on teachers, undermining their ability to teach effectively and, in turn, weakening perceptions of management commitment and support, as well as management priority.

Findings also reinforced the need for leaders to manage pedagogical pressures, which is consistent with recent literature that emphasises the significance of these challenges in educational settings. For instance, a report highlighted that 15–20% of Aotearoa New Zealand’s students are neurodivergent, yet only 6–7% of students receive public funding for learning support, which strains teachers, who work “well beyond their role trying to support these kids” [72] (p. 23). Hence, leaders who understand the pedagogical pressures teachers face, enables them to respond swiftly and, where possible, employ interventions that support learning and, in turn, teachers’ psychological H&S. For example, at the start of the scholastic year, a line manager might go through the class register with a beginning teacher identifying the learning needs of individual students and co-developing personalised learning programmes for those at greatest risk of underachievement. This enables the development of effective interventions, which, as Loh et al. [9] claimed, are often accompanied by job resources that improve working conditions. The extension the present research offers is that school leaders need to be knowledgeable and skilled in managing pedagogical pressures before they can design and implement effective policies and procedures that safeguard teachers’ psychological H&S.

While PSC is a leading indicator of better working conditions, resulting in manageable demands and adequate resources [73], the present research found that possessing empathy and communication skills enables leaders to facilitate organisational communication and participation. Therefore, leaders’ interpersonal skills may facilitate participation in PLCs, teacher–leader mentoring sessions and staff meetings, as these leaders are better at sharing ideas and making teachers feel psychologically safe. While organisational communication is critical for enhancing organisational participation and promoting employee psychological H&S [6], the present investigation underlines that interpersonal skills encourage upward communication even in challenging situations. Although recent evidence suggests that empathy and listening amongst teacher leaders could be pivotal to building PSC by prioritising teacher welfare and development [43], the present research extends this work by demonstrating that these interpersonal skills are potentially more important for leaders with substantial line management responsibilities, given their greater discretion and authority to utilise them when implementing policies, practices, and procedures.

Participants reported that leaders who are receptive to suggestions and concerns are more mindful of the challenges staff face and are more likely to act promptly. Literature has also shown that in positive PSC environments, leaders are more likely to manage situations and take swift action when acute problems arise [7,45]. The current research deepens this understanding in school settings by showing that teachers value leaders not only for their awareness of working conditions but also for having clear and effective policies and procedures in place to prevent or mitigate psychosocial hazards. This emphasis is consistent with the literature suggesting that preventive measures are more effective than reactive efforts that modify working conditions or address the emotional impact once it manifests [74]. Thus, teachers appreciated leaders who are skilled in devising robust policies and procedures that anticipate or minimise the impact of incidents. These findings advance existing knowledge by demonstrating that proactive leadership behaviours play a central, previously under-examined role in reinforcing PSC in school settings.

Finally, this research shows that pedagogical expertise is another essential leadership competency, as it may enable management commitment and support, and organisational communication through timely pedagogical interventions. School leaders are often viewed by participants as experts in pedagogy, which they can use effectively to mentor and coach teachers who may need support in various aspects of their roles. Pedagogical expertise may include motivating students, applying a variety of learning strategies, possessing strong classroom management skills, and differentiating lessons [75], all of which can take time to acquire, especially for a beginning teacher. When leaders openly share valuable skills and knowledge with staff and implement effective teaching strategies, this expertise may not only put teachers at ease but also address job demands. The sharing of knowledge between leaders and employees is a job resource because it can mitigate risks associated with unexpected work peaks or emotional demands [38,76]. This is particularly the case in teaching, whereby a leader may mitigate these risks by discussing suitable assessment strategies for specific courses and students.

### 6.1. The Development of a Leader Competence–PSC Framework

**Figure 1:**
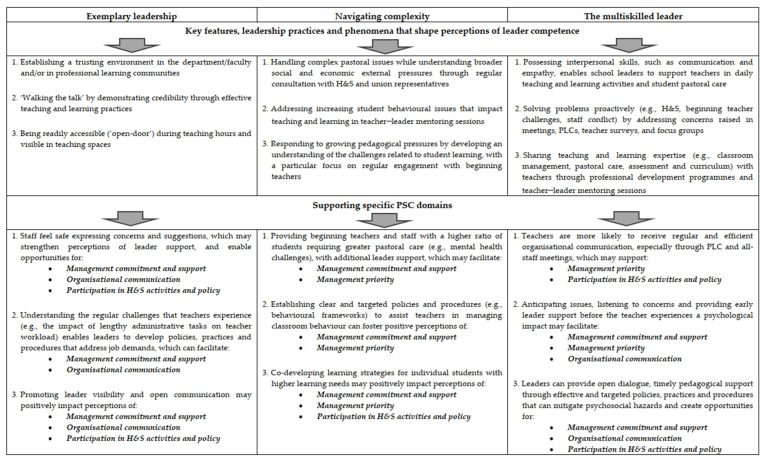
Leader Competence–PSC Framework.

Based on the findings explained above, we propose the Leader Competence–PSC Framework (Figure 1). The framework illustrates that the three leadership competency indicators (*exemplary leadership*, *navigating complexity*, and *multiskilled leadership*) may facilitate the emergence of key features, leadership practices and phenomena (associated subthemes) that can enable PSC implementation in an educational context. Each leadership competency is then explicitly linked with the four domains of the PSC framework. The arrows indicate impact, while the numbers located in the upper quadrants correspond to the numbers in the lower quadrants, located directly below.

The framework is presented as a practical tool for schools seeking to evaluate and improve PSC. For example, schools could utilise the framework for training/guiding school principals and other leaders on which areas of competence require specific attention and are likely to have positive implications for PSC. This framework could also provide school leaders with relevant guidance on how leadership competence can enable the implementation of PSC that specifically protects teachers’ psychological H&S.

The Leader Competence–PSC Framework can be employed as a planning and development tool. It highlights the interchange between specific school leader competencies and PSC domains, enabling schools to identify gaps in leadership capability and inform the design of professional learning programmes. For example, training could focus on strategies and interventions for managing pastoral and behavioural issues, as well as on practices that enhance exemplary leadership behaviours, such as building trust and accessibility. Additionally, it can support mentoring and coaching by providing tangible skills and behaviours that leaders can apply in everyday settings. Embedding this framework in induction programmes and ongoing appraisal systems could also help schools create a culture in which teacher psychological H&S is systematically addressed and prioritised through leadership practice.

The Leader Competence–PSC Framework, together with the findings in the discussion section, provide rich contextual insights into specific leadership competencies related to PSC in an educational setting. The examination of these competencies has shed light on how each one can facilitate PSC implementation across the four domains of the PSC framework. This approach differs from most extant literature in two key ways. First, it offers a deeper, more context-specific interpretation of leadership in relation to PSC than the extant quantitative literature. Second, while the literature has primarily examined the impact of leadership styles on PSC [8,9,10,11], the present research demonstrates that PSC implementation is effective when leaders possess the specific competencies that can yield practical and meaningful PSC outcomes.

### 6.2. Contributions and Practical Implications

This investigation makes notable practical and theoretical contributions. The theoretical contribution is the conceptualisation of leadership competence as a novel and complementary lens for understanding how leadership can enable PSC implementation within an educational setting. The relationship between leadership competence and PSC implementation highlights the specific skills, knowledge, and behaviours critical for effective PSC development. This research advances understanding and clarifies how actionable school leader competencies, such as handling pastoral issues, addressing misbehaviour, and managing pedagogical pressures, can enhance teachers’ job resources, reduce job demands, and potentially improve and sustain perceptions of a positive PSC environment. As noted above, the Leader Competence–PSC Framework (Figure 1) illustrates how these competencies are applied in leaders’ everyday tasks and responsibilities and are linked to individual PSC domains. While an increasing number of studies have demonstrated that leadership is a critical factor in shaping PSC [8,9,10,11], most of this work has focused on the influence of different leadership styles. This approach tends to overlook the importance of contextual features essential to developing a deeper understanding of how leadership shapes PSC in an educational workplace and, more generally, presents a one-dimensional account of leadership. In contrast, the present research is part of a limited body of literature that offers an alternative, qualitatively informed perspective on how leadership enables PSC implementation in a challenging, continuously evolving school environment.

### 6.3. Limitations and Future Research

Although case studies provide rich, contextual insights [50], statistical generalisability is not their aim. Our research, however, provides analytical generalisability [50] in relation to relevant PSC and leadership theory. Additionally, by providing a rich contextual description, we enable readers to assess the applicability of the findings to comparable educational contexts [77]. The likelihood of transferability of this study’s findings is supported by the literature [78,79], which indicates that schools, teachers, and students across diverse educational systems face similar challenges and pressures, suggesting that in educational settings with comparable conditions, the leadership competencies that support and sustain PSC implementation examined in this study may also be relevant.

As a recommendation for future research, it would be interesting to conduct rigorous longitudinal investigations using the Leader Competence–PSC Framework (Figure 1, p. 29) to examine how shifts in leadership capability influence PSC over time. For example, quantitative research could assess the relationship between the levels of the identified competencies that school leaders possess and the PSC of their work group. Additionally, qualitative research could extend the analytical generalisability of the present findings by being conducted across a wide range of educational contexts. More generally, applying the framework in contexts beyond education could yield valuable insights.

## 7. Conclusions

This study provides novel insights into the impact that leader competence has on PSC implementation. More precisely, it shows how exemplary leadership, navigating complexity, and multiskilled leadership enable school leaders to design and implement policies, practices and procedures that develop PSC across its four domains. The Leader Competence–PSC Framework (Figure 1, p. 29) depicts how each leadership competence indicator impacts individual PSC domains. The framework provides practical suggestions and insights for improving leadership competence and, in turn, PSC implementation. Further, this framework can improve the selection and recruitment process for leadership roles in selecting candidates who demonstrate proficiency in exercising these competencies or the ability to develop them. Lastly, we advocate using this framework to help focus future research in this area.

## Figures and Tables

**Table 1:** Participant demographic.

Gender	Female: 17					Male: 9				
**Age**	20–29: 4	30–39: 5			40–49: 8		50–59: 5			>60: 3
**Ethnicity/nationality** (may have identified with more than one ethnicity/nationality)	Māori: 2					NZ European: 12				
	Chinese: 1									
	Fijian: 2					Indian: 7				
	Samoan: 1					Another ethnicity/				
	South African: 3					nationality: 4				
**Time employed at school**	0–3 years: 6			4–10 years: 11				More than 10 years: 9		
**Participants/representation from each team** (excludes principal and support staff)	Team A: 6		Team B: 6			Team C: 6			Team D: 5	
**Years employed as a teacher in total** (includes previous employment in schools)	1–3 years: 3		4–10 years: 5			11–20 years: 6			>20 years: 12	
**Employment agreement**	Full-time: 23					Part-time: 3				
**Average hours spent completing work per week** (full-time staff only)	46 h									

## Data Availability

We are currently in possession of all interview and focus group transcripts/audio recordings. However, these are not in a public depository but in the authors’ possession.
